# Hahn Sequence Space of Modals

**DOI:** 10.1155/2014/974934

**Published:** 2014-11-09

**Authors:** T. Balasubramanian, S. Zion Chella Ruth

**Affiliations:** ^1^Department of Mathematics, Kamaraj College, Tuticorin, Tamilnadu 628003, India; ^2^Department of Mathematics, Dr. G. U. Pope College of Engineering, Sawyerpuram, Tuticorin, Tamilnadu 628251, India

## Abstract

The history of modal intervals goes back to the very first publications on the topic of interval calculus. The modal interval analysis is used in Computer graphics and Computer Aided Design (CAD), namely, the computation of narrow bounds on Bezier and *B*-Spline curves. Since modal intervals are used in many fields, we introduce a new sequence space *h(gI)* called the Hahn sequence space of modal intervals. We have given some new definitions and theorems. Some inclusion relation and some topological properties of this space are investigated. Also dual spaces of this space are computed.

## 1. Introduction

Interval arithmetic was first suggested by Dwyer [1] in 1951. Furthermore, Moore and Yang [2, 3] have developed applications to differential equations. Chiao in 2002 [4] introduced sequence of interval numbers and defined usual convergence of sequences of interval number. Recently, Zararsız and Şengönül [5] introduced null, bounded, and convergent sequence space of modals. Hahn in 1922 [6] defined *h* space and G. Goes and S. Goes [7] in 1970 studied the functional analytic properties of this space. The Hahn sequence space was initiated by Rao in 1990 [8]. The present paper is devoted to the study of Hahn sequence space of modal intervals.

Let us denote the set of all real valued closed interval by *I*, the set of positive integers by *N*, and the set of all real numbers by *ℜ*. Any element of *I* is called interval number and it is denoted by $\hat{x}$. That is $\hat{x}=\{x\in ℜ:\underline{x}\leq x\leq \overset{-}{x}\}$. An interval number $\hat{x}$ is a closed subset of real numbers. Let $\underline{x}$ and $\overset{-}{x}$ be, respectively, first and last points of the interval number $\hat{x}$. Therefore, when $\underline{x}>\overset{-}{x}$, $\hat{x}$ is not an interval number. But in modal analysis $\left[\overset{-}{x},\underline{x}\right]$ is a valid interval. A modal $\tilde{x}=\{[\underline{x},\overset{-}{x}]:\underline{x},\overset{-}{x}\in ℜ\}$ is defined by a pair of real numbers $\overset{-}{x},\underline{x}$. Let us denote the set of all modals by *gI*. Let us suppose that $\tilde{x},\tilde{y}\in gI$. Then the algebraic operations between $\tilde{x}$ and $\tilde{y}$ are defined in the Kaucher arithmetic [9]. For a modal $\tilde{x}=[\underline{x},\overset{-}{x}]\text{dual}$ operator is defined as $\text{dual}\tilde{x}=[\overset{-}{x},\underline{x}]$. Thus, if $\tilde{x}\in gI$, then $\tilde{x}-\text{dual}\tilde{x}=[0,0]=\tilde{0}$, $\text{dual}\tilde{x}\in gI$. Let us suppose that $\tilde{x}\in gI$; then $\tilde{x}$ is called symmetric modal if $\underline{x}=-\overset{-}{x}$ or vice versa.

The set of all modals *gI* is a metric space with the metric *d* defined by

(1) $$\begin{array}{c} d\left({\tilde{x}}_{1},{\tilde{x}}_{2}\right)=\max\left\{\left|\underline{x_{1}}-\underline{x_{2}}\right|,\left|{\overset{-}{x}}_{1}-{\overset{-}{x}}_{2}\right|\right\}. \end{array}$$

If $\tilde{x},\tilde{y}\in gI$ and $\underline{x}\leq \overset{-}{x}$, $\underline{y}\leq \overset{-}{y}$ then the set *gI* is reduced ordinary set of interval numbers which is complete metric space with the metric *d* defined in (1) [4]. If we take ${\tilde{x}}_{1}=[a,a]$ and ${\tilde{x}}_{2}=[b,b]$, we obtain the usual metric of *ℜ* with $d({\tilde{x}}_{1},{\tilde{x}}_{2})=\left|a-b\right|$, where *a*, *b* ∈ *ℜ*.

## 2. Definitions and Preliminaries

Let *f* be a function from *N* to *gI* which is defined by $k\to f(k)=\tilde{x}$, $\tilde{x}=({\tilde{x}}_{k})$. Then $\left({\tilde{x}}_{k}\right)$ is called sequence of modals. We will denote the set of all sequences of modals by *w*(*gI*).

For two sequences of modals $\left({\tilde{x}}_{k}\right)$ and $\left({\tilde{y}}_{k}\right)$, the addition, scalar product, and multiplication are defined as follows: $\left({\tilde{x}}_{k}+{\tilde{y}}_{k})=[{\underline{x}}_{k}+{\underline{y}}_{k},{\overset{-}{x}}_{k}+{\overset{-}{y}}_{k}\right]$, $\left(\alpha {\tilde{x}}_{k})=[\alpha {\underline{x}}_{k},\alpha {\overset{-}{x}}_{k}\right]$, *α* ∈ *ℜ*, $\left({\tilde{x}}_{k}{\tilde{y}}_{k})=[\min({\underline{x}}_{k}{\underline{y}}_{k},{\underline{x}}_{k}{\overset{-}{y}}_{k},{\overset{-}{x}}_{k}{\underline{y}}_{k},{\overset{-}{x}}_{k}{\overset{-}{y}}_{k}\right)$,$\;\max({\underline{x}}_{k}{\underline{y}}_{k},{\underline{x}}_{k}{\overset{-}{y}}_{k},{\overset{-}{x}}_{k}{\underline{y}}_{k},{\overset{-}{x}}_{k}{\overset{-}{y}}_{k})]$, respectively.

The set *w*(*gI*) is a vector space since the vector space rules are clearly provided. The zero element of *w*(*gI*) is the sequence $\tilde{\theta }=({\tilde{\theta }}_{k})=([0,0])$, all terms of which are zero interval. If $\left({\tilde{x}}_{k})\in w(gI\right)$ then inverse of $\left({\tilde{x}}_{k}\right)$, according to addition, is $\text{dual}({\tilde{x}}_{k})$.

Let *λ*(*gI*) ⊂ *w*(*gI*). If a sequence space contains a sequence $\left({\tilde{e}}_{n}\right)$ of modals with the property that for every $\tilde{u}\in \lambda (gI)$ there is a unique sequence of scalars $\left({\tilde{t}}_{n}\right)$ such that $\lim_{n}d(\tilde{u},{\tilde{t}}_{1}{\tilde{e}}_{1}+\cdots +{\tilde{t}}_{n}{\tilde{e}}_{n})\to \tilde{0}$ then $\left({\tilde{e}}_{n}\right)$ is called a Schauder modal basis for *λ*(*gI*). The series $\sum_{k=1}^{\infty }{\tilde{t}}_{k}{\tilde{e}}_{k}$ which has the sum $\tilde{u}$ is then called the expansion of $\tilde{u}$ with respect to $\left({\tilde{e}}_{n}\right)$, and we write $\tilde{u}=\sum_{k=1}^{\infty }{\tilde{t}}_{k}{\tilde{e}}_{k}$.

Let *λ*(*gI*) and *μ*(*gI*) be linear space of modals. Then a function $\tilde{A}:\lambda (gI)\to \mu (gI)$ is called a linear transformation if and only if, for all ${\tilde{u}}_{1},{\tilde{u}}_{2}\in \lambda (gI)$ and all ${\tilde{t}}_{1},{\tilde{t}}_{2}\in gI$, $\tilde{A}({\tilde{t}}_{1}{\tilde{u}}_{1}+{\tilde{t}}_{2}{\tilde{u}}_{2})={\tilde{t}}_{1}\tilde{A}{\tilde{u}}_{1}+{\tilde{t}}_{2}\tilde{A}{\tilde{u}}_{2}$.

**Proposition 1 .**

If $\left({\tilde{x}}_{k}\right)$, $\left({\tilde{y}}_{k}\right)$, and $\left({\tilde{r}}_{k}\right)$ are sequences of symmetric modal, then the following equality holds:

(2) $$\begin{array}{c} \left({\tilde{x}}_{k}\right)\left\{\left({\tilde{y}}_{k}\right)-\left({\tilde{r}}_{k}\right)\right\}=\left({\tilde{x}}_{k}\right)\left({\tilde{y}}_{k}\right)-\left({\tilde{x}}_{k}\right)\left({\tilde{r}}_{k}\right). \end{array}$$

**Definition 2 .**

A sequence $\tilde{x}=({\tilde{x}}_{k})\in w(gI)$ of modals is said to be convergent to the modal ${\tilde{x}}_{0}$ if for each *ε* > 0 there exists a positive integer *n*_0_ such that $d({\tilde{x}}_{k},{\tilde{x}}_{0})<\varepsilon$ for all *k* ≥ *n*_0_ and we denote it by writing $\lim_{k}{\tilde{x}}_{k}={\tilde{x}}_{0}$. Thus, $\lim_{k\to \infty }{\tilde{x}}_{k}={\tilde{x}}_{0}\Leftrightarrow \lim_{k\to \infty }{\underline{x}}_{k}={\underline{x}}_{0}$ and $\lim_{k\to \infty }{\overset{-}{x}}_{k}={\overset{-}{x}}_{0}$.

**Definition 3 .**

A sequence of modals, $\tilde{x}=({\tilde{x}}_{k})\in w(gI)$, is said to be modal fundamental sequence if for every *ε* > 0 there exists *k*_0_ ∈ *N* such that $d({\tilde{x}}_{k},{\tilde{x}}_{n})<\varepsilon$ whenever *n*, *k* > *k*_0_.

**Definition 4 .**

A sequence of modals *w*(*gI*) is said to be solid if $\tilde{y}=({\tilde{y}}_{k})\in w(gI)$ whenever $||{\tilde{y}}_{k}||\leq ||{\tilde{x}}_{k}||$ for all *k* ∈ *N* and $\tilde{x}=({\tilde{x}}_{k})\in w(gI)$.

**Definition 5 .**

A sequence of generalized intervals *w*(*gI*) is said to be monotone if *w*(*gI*) contains the canonical preimage of all its step spaces.

**Definition 6 (Weierstrass *M*-test).**

Let ${\tilde{f}}_{k}:gI\to gI$ be given. If there exists an *M*_*k*_ ≥ 0 such that $d[{\tilde{f}}_{k}(\tilde{x}),\tilde{0}]\leq M_{k}$ and the series ∑_*k*=1_^*∞*^ *M*_*k*_ converges, then the series $\sum_{k=1}^{\infty }{\tilde{f}}_{k}(\tilde{x})$ is uniformly and absolutely convergent in *gI*.

The following spaces are needed for our work:

(3) c0gI=u~=u~k∈wgI:lim⁡k→∞du~k,0~=0cgI=u~=u~k∈wgI:lim⁡k→∞du~k,u~0=0l∞gI=u~=u~k∈wgI:sup⁡kdu~k,0~<∞csgI=u~=u~k∈wgI:lim⁡n→∞d∑k=0nu~k,u~0=0bs(gI)=u~=u~k∈wgI:sup⁡nd∑k=1nu~k,0~<∞l1gI=u~=u~k∈wgI:∑k=1∞du~k,0~<∞bvgI=u~=u~k∈wgI:∑k=1∞du~k−u~k+1,0~<∞bv0gI=u~=u~k∈wgI:∑k=1∞du~k−u~k+1,0~<∞,  lim⁡k→∞d(u~k,0~)=0u~=u~k∈wgI:∑k=1∞du~k−u~k+1,0~<∞,σ∞(gI)=u~=u~k∈wgI:sup⁡n1nd∑k=1nu~k,0~<∞∫E(gI)=u~=u~k∈wgI:ku~k∈EgIhhhhhhhhhhhis  the  integrated  space  of  E(gI)dEgI=u~=u~k∈wgI:1ku~k∈EgIhhhhhhhhhis  the  differentiated  space  of  EgI.

## 3. Main Results

Define a sequence $\tilde{y}=({\tilde{y}}_{k})$ which will be frequently used as the *T*-transform of a sequence $\tilde{x}=({\tilde{x}}_{k})\in w(gI)$. That is, ${\tilde{y}}_{k}=(T\tilde{x}{)}_{k}=k({\tilde{x}}_{k}-{\tilde{x}}_{k+1})$, *k* ≥ 1.

We introduce the sequence spaces *h*(*gI*) and *h*_*∞*_(*gI*) as the set of all sequences such that *T*-transforms of them are in *l*(*gI*).

That is,

(4) hgI=u~=(u~k)∈w(gI):∑k=1∞dTu~k,0~<∞,hhhlim⁡k→∞du~k,0~=0u~=u~k∈wgI:∑k=1∞dTu~k,0~<∞,,h∞gI=u~=u~k∈wgI:sup⁡kdTu~k,0~<∞.

*h*(*gI*) is a normed space with the norm $\left\|\tilde{u}\right\|=\sum_{k=1}^{\infty }d((T\tilde{u}{)}_{k},\tilde{0})$.

**Example 7 .**

Consider the sequence $\tilde{u}=({\tilde{u}}_{k})$ defined by

(5) $$\begin{array}{c} \tilde{u}=\left({\tilde{u}}_{k}\right)=\left\{\begin{array}{cc} \left[1,1\right], & 1\leq k\leq n\\ \left[0,0\right], & k>n. \end{array}\right. \end{array}$$

Note that $\sum_{k=1}^{\infty }d((T\tilde{u}{)}_{k},\tilde{0})=\sum_{k=1}^{\infty }d(k({\tilde{u}}_{k}-{\tilde{u}}_{k+1}),\tilde{0})=0$ which is convergent.

Also $\lim_{k\to \infty }d({\tilde{u}}_{k},\tilde{0})=0$.

Hence $\tilde{u}=({\tilde{u}}_{k})\in h(gI)$.

**Theorem 8 .**

*h*(*gI*) and *h*_*∞*_(*gI*) are complete metric spaces with the metrics ${\tilde{d}}_{h}$ and ${\tilde{d}}_{h_{\infty }}$ defined by

(6) $$\begin{array}{c} {\tilde{d}}_{h}\left(\tilde{u},\tilde{v}\right)=\overset{\infty }{\underset{k=1}{\sum }}d\left[{\left(T\tilde{u}\right)}_{k},{\left(T\tilde{v}\right)}_{k}\right], \end{array}$$

(7) $$\begin{array}{c} {\tilde{d}}_{h_{\infty }}\left(\tilde{u},\tilde{v}\right)=\underset{k}{\sup}d\left[{\left(T\tilde{u}\right)}_{k},{\left(T\tilde{v}\right)}_{k}\right], \end{array}$$

respectively, where $\tilde{u}=({\tilde{u}}_{k})$ and $\tilde{v}=({\tilde{v}}_{k})$ are the elements of the space *h*(*gI*) or *h*_*∞*_(*gI*).

**Proof**

Let $\left\{{\tilde{u}}^{\left(i\right)}\right\}$ be any fundamental sequence in the space *h*(*gI*), where $\left\{{\tilde{u}}^{\left(i\right)}\}=\{{{\tilde{u}}_{0}}^{\left(i\right)},{{\tilde{u}}_{1}}^{\left(i\right)},\ldots \right\}$. Then for a given *ε* > 0, there exists a positive integer *n*_0_(*ε*) such that

(8) d~hu~i,u~j=∑k=1∞dTu~ik,Tu~jk<ε,hhhhhhhhhhhhhhhhhhhhhhhh∀i,j≥n0ε.

We obtain for each fixed *k* ∈ *N* from (8) that

(9) $$\begin{array}{c} d\left[{\left(T{\tilde{u}}^{\left(i\right)}\right)}_{k},{\left(T{\tilde{u}}^{\left(j\right)}\right)}_{k}\right]<\varepsilon \;\text{for}\;\text{every}\;i,j\geq n_{0}\left(\varepsilon \right)\; \end{array}$$

which leads to the fact that $\left\{(T{\tilde{u}}^{\left(i\right)}{)}_{k}\right\}$ is a fundamental sequence in *gI* for every fixed *k* ∈ *N*.

Since *gI* is complete, $\left\{(T{\tilde{u}}^{\left(i\right)}{)}_{k}\right\}$ converges to $(T\tilde{u}{)}_{k}$ as *i* → *∞*.

Consider the sequence $\left\{(T\tilde{u}{)}_{1},(T\tilde{u}{)}_{2},\ldots \right\}$; we have from (9) for each *m* ∈ *N* and *i*, *j* ≥ *n*_0_(*ε*) that

(10) ∑k=1mdTu~ik,Tu~jk≤d~hu~i,u~j<ε,hhhhhhhhhhhhhhhhhhhhhhhfor  i,j≥n0ε.

Take any *i* ≥ *n*_0_(*ε*) and take limit as *j* → *∞* first and then let *m* → *∞* in (8); we obtain

(11) $$\begin{array}{c} {\tilde{d}}_{h}\left({\tilde{u}}^{\left(i\right)},\tilde{u}\right)<\varepsilon . \end{array}$$

Therefore ${\tilde{u}}^{\left(i\right)}\to \tilde{u}$.

We have to prove $\tilde{u}\in h(gI)$.

Since $\left\{{\tilde{u}}^{\left(i\right)}\right\}$ is a fundamental sequence in *h*(*gI*), we have

(12) $$\begin{array}{c} \overset{\infty }{\underset{k=1}{\sum }}d\left({\left(T{\tilde{u}}^{\left(i\right)}\right)}_{k},\tilde{0}\right),\;\;\underset{k\to \infty }{\lim}d\left({\tilde{u}}_{k},\tilde{0}\right)=0. \end{array}$$

Now,

(13) dTu~k,0~≤dTu~k,Tu~ik+dTu~ik,Tu~jk +dTu~jk,0~.

Hence

(14) ∑k=1∞dTu~k,0~≤∑k=1∞dTu~k,Tu~ik +∑k=1∞dTu~ik,Tu~jk +∑k=1∞dTu~jk,0~.

Also from (10) and (11), $\lim_{k\to \infty }d[(T\tilde{u}{)}_{k},\tilde{0})=0$. Hence $\tilde{u}\in h(gI)$. Since $\left\{{\tilde{u}}^{\left(i\right)}\right\}$ is an arbitrary fundamental sequence, the space *h*(*gI*) is complete.

Similarly, we can prove *h*_*∞*_(*gI*) is complete space.

**Theorem 9 .**

The space *h*(*gI*) is monotone.

**Proof**

Let *n* < *m*; it follows from

(15) du~n,0~≤du~n−u~n+1,0~+du~n+1−u~n+2,0~ +⋯+du~m−1−u~m,0~+du~m,0~

that

(16) u~(n)≤∑k=1n−1kdu~k−u~k+1,0~+ndu~n−u~n+1,0~ +m−1du~m−1−u~m,0~+mdu~m,0~=u~m.

The sequence $\left(||{\tilde{u}}^{\left(n\right)}||\right)$ is monotone increasing; it thus follows from $\tilde{u}=\lim_{n\to \infty }{\tilde{u}}^{\left(n\right)}$ that $\left\|\tilde{u}\right\|=\lim_{n\to \infty }||{\tilde{u}}^{\left(n\right)}||=\sup_{n}||{\tilde{u}}^{\left(n\right)}||$. This completes the proof.

**Theorem 10 .**

The space *h*(*gI*) is not rotund.

**Proof**

Consider the sequences $\tilde{u}=({\tilde{u}}_{k})$ and $\tilde{v}=({\tilde{v}}_{k})$ defined by

(17) $$\begin{array}{c} \tilde{u}=\left({\tilde{u}}_{k}\right)=\left\{\left[1,1\right],\tilde{0},\tilde{0},\ldots \right\},\\ \tilde{v}=\left({\tilde{v}}_{k}\right)=\left\{\left[1/2,1/2,\right],\left[1/2,1/2,\right],\tilde{0},\tilde{0},\ldots \right\}. \end{array}$$

Then

(18) $$\begin{array}{c} \overset{\infty }{\underset{k=1}{\sum }}d\left({\left(T\tilde{u}\right)}_{k},\tilde{0}\right)<\infty ,\;\;\overset{\infty }{\underset{k=1}{\sum }}d\left({\left(T\tilde{v}\right)}_{k},\tilde{0}\right)<\infty . \end{array}$$

Also,

(19) $$\begin{array}{c} \underset{k\to \infty }{\lim}d\left[{\tilde{u}}_{k},\tilde{0}\right]=0,\;\;\underset{k\to \infty }{\lim}d\left[{\tilde{v}}_{k},\tilde{0}\right]=0. \end{array}$$

Thus $\tilde{u}=({\tilde{u}}_{k})$ and $\tilde{v}=({\tilde{v}}_{k})$ are in *h*(*gI*) and ${\tilde{d}}_{h}(\tilde{u},\tilde{0})={\tilde{d}}_{h}(\tilde{v},\tilde{0})=1$.

Note that $\tilde{u}\neq \tilde{v}$, but ${\tilde{d}}_{h}(\left(\tilde{u}+\tilde{v}\right)/2,\tilde{0})=1$. Therefore *h*(*gI*) is not rotund.

**Theorem 11 .**

The space *h*(*gI*) is not solid.

**Proof**

Consider the sequence

(20) $$\begin{array}{c} \tilde{u}=\left({\tilde{u}}_{k}\right)=\left\{\begin{array}{cc} \left[1,1\right], & 1\leq k\leq n\\ \left[0,0\right], & k>n, \end{array}\right.\\ \tilde{v}=\left({\tilde{v}}_{k}\right)=\left(\left[{\left(-1\right)}^{k},{\left(-1\right)}^{k}\right]\right). \end{array}$$

Since

(21) $$\begin{array}{c} d\left[{\tilde{v}}_{k},\tilde{0}\right]=d\left[{\tilde{u}}_{k},\tilde{0}\right]=1,\;\;d\left({\left(T\tilde{u}\right)}_{k},\tilde{0}\right)=0, \end{array}$$

it immediately follows that $\tilde{u}=({\tilde{u}}_{k})\in h(gI)$.

However, it is trivial that $d((T\tilde{v}{)}_{k},\tilde{0})=2$. $d((T\tilde{v}{)}_{k},\tilde{0})≰d((T\tilde{u}{)}_{k},\tilde{0})$, which implies $\tilde{v}=({\tilde{v}}_{k})\notin h(gI)$. This completes the proof.

## 4. Dual Space of *h*(*gI*)

**Definition 12 .**

The *α*-dual, *β*-dual, and *γ*-dual of *s*(*gI*) ⊂ *w*(*gI*) are, respectively, defined by

(22) sgIα =u~k∈wgI:u~kv~k∈l1gI  ∀v~k∈sgIsgIβ =u~k∈wgI:u~kv~k∈csgI  ∀v~k∈sgIsgIγ =u~k∈wgI:u~kv~k∈bsgI  ∀v~k∈sgI.

It is trivial that the following inclusions hold: {*s*(*gI*)}^*α*^ ⊂ {*s*(*gI*)}^*β*^ ⊂ {*s*(*gI*)}^*γ*^.

**Theorem 13 .**

Let *E*(*gI*) and *E*_1_(*gI*) be the sets of sequences of modals. Then the following statements hold.

(i) *E*(*gI*)⊂[*E*(*gI*)]^*ββ*^.
(ii) [*E*(*gI*)]^*βββ*^ = [*E*(*gI*)]^*β*^.
(iii) If *E*_1_(*gI*)⊃*E*(*gI*) then [*E*_1_(*gI*)]^*β*^ ⊂ [*E*(*gI*)]^*β*^.

The same results hold for dual also.

**Proof**

Let $\tilde{u}=({\tilde{u}}_{k})$, $\tilde{v}=({\tilde{v}}_{k})$, and $\tilde{w}=({\tilde{w}}_{k})$ be sequences of modal intervals.

(i) Suppose $\tilde{u}=({\tilde{u}}_{k})\notin [E(gI){]}^{\beta \beta }$; then $\left({\tilde{u}}_{k}{\tilde{v}}_{k})\notin cs(gI\right)$ for at least one $\tilde{v}=({\tilde{v}}_{k})\in [E(gI){]}^{\beta }$. But $\tilde{v}=({\tilde{v}}_{k})\in [E(gI){]}^{\beta }$ implies that $\left({\tilde{u}}_{k}{\tilde{v}}_{k})\in cs(gI\right)$ for all $\tilde{w}=({\tilde{w}}_{k})\in E(gI)$.

This means that $\tilde{u}$ is not a member of *E*(*gI*). Hence *E*(*gI*)⊂[*E*(*gI*)]^*ββ*^. The proof for (ii) follows similarly.

(iii) Suppose $\tilde{u}=({\tilde{u}}_{k})\in [E_{1}(gI){]}^{\beta }$; then $\left({\tilde{u}}_{k}{\tilde{v}}_{k})\in cs(gI\right)$ for all $\tilde{v}=({\tilde{v}}_{k})\in E_{1}(gI)$. Since *E*_1_(*gI*)⊃*E*(*gI*), $\tilde{v}=({\tilde{v}}_{k})\in E(gI)$. Thus [*E*_1_(*gI*)]^*β*^ ⊂ [*E*(*gI*)]^*β*^.

Define the sequence $\tilde{y}=({\tilde{y}}_{k})$ which will be frequently used as the *B*-transform of a sequence $\tilde{x}=({\tilde{x}}_{k})\in w(gI)$.

That is,

(23) $$\begin{array}{c} {\tilde{y}}_{k}={\left(B\tilde{x}\right)}_{k}=\frac{1}{k}\overset{k}{\underset{i=1}{\sum }}{\tilde{x}}_{i}. \end{array}$$

The Cesaro space of *l*_*∞*_(*gI*) is the set of all sequences such that the *B*-transforms of them are in *l*_*∞*_(*gI*). That is, $\sigma (l_{\infty }(gI))=\{\tilde{x}=({\tilde{x}}_{k}),\sup_{k}d[{\left(B\tilde{x}\right)}_{k},\tilde{0}]<\infty \}$.

**Theorem 14 .**

*σ*(*l*_*∞*_(*gI*)) is a complete metric space with the metric ${\tilde{d}}_{\sigma }(\tilde{u},\tilde{v})=\sup_{k}d[{\left(B\tilde{u}\right)}_{k},{\left(B\tilde{v}\right)}_{k}]$ where $\tilde{u}=({\tilde{u}}_{k})$ and $\tilde{v}=({\tilde{v}}_{k})$ are the elements of space *σ*(*l*_*∞*_(*gI*)).

**Proof**

Let $\left\{{\tilde{u}}^{\left(i\right)}\right\}$ be any fundamental sequence in the space *σ*(*l*_*∞*_(*gI*)) where $\left\{{\tilde{u}}^{\left(i\right)}\}=\{{{\tilde{u}}_{0}}^{\left(i\right)},{{\tilde{u}}_{1}}^{\left(i\right)},\ldots \right\}$. Then for a given *ε* > 0, there exists a positive integer *n*_0_(*ε*) such that

(24) d~σu~i,u~j=sup⁡kdBu~ik,Bu~jk<ε,hhhhhhhhhhhhhhhhhhhhhhhhh∀i,j≥n0ε.

We obtain for each fixed *k* ∈ *N* from (24) that

(25) $$\begin{array}{c} d\left[{\left(B{\tilde{u}}^{\left(i\right)}\right)}_{k},{\left(B{\tilde{u}}^{\left(j\right)}\right)}_{k}\right]<\varepsilon \end{array}$$

which leads to the fact that $(B{\tilde{u}}^{\left(i\right)}{)}_{k}$ is a fundamental sequence for every fixed *k* ∈ *N*. Since *gI* is complete, $(B{\tilde{u}}^{\left(i\right)}{)}_{k}\to (B\tilde{u}{)}_{k}$ as *i* → *∞*.

Consider the sequence $\left\{(B\tilde{u}{)}_{1},(B\tilde{u}{)}_{2},\ldots \right\}$. We have from (25) for each *m* ∈ *N* and *i*, *j* ≥ *n*_0_(*ε*) that

(26) sup⁡kdBu~ik,Bu~jk≤d~σu~i,u~j<ε,hhhhhhhhhhhhfor  k=1,2…m,  ∀i,j≥n0ε.

For any *i* ≥ *n*_0_(*ε*), taking limit *j* → *∞* first and letting *m* → *∞* in (24), we obtain

(27) $$\begin{array}{c} {\tilde{d}}_{\sigma }\left({\tilde{u}}^{\left(i\right)},\tilde{u}\right)<\varepsilon . \end{array}$$

Finally, we proceed to prove $\tilde{u}\in \sigma (l_{\infty }(gI))$. Since $\left\{{\tilde{u}}^{\left(i\right)}\right\}$ is a fundamental sequence in *σ*(*l*_*∞*_(*gI*)), we have $\sup_{k}d[(B{\tilde{u}}^{\left(i\right)}{)}_{k},\tilde{0}]<\infty$.

Now,

(28) dBu~k,0~≤dBu~k,Bu~ik +dBu~ik,Bu~jk+dBu~jk,0~,sup⁡kdBu~k,0~≤sup⁡kdBu~k,Bu~ik +sup⁡kdBu~ik,Bu~jk +sup⁡kdBu~jk,0~<∞.

Hence $\tilde{u}\in \sigma (l_{\infty }(gI))$.

Since $\left\{{\tilde{u}}^{\left(i\right)}\right\}$ is an arbitrary fundamental sequence, the space *σ*(*l*_*∞*_(*gI*)) is complete.

**Theorem 15 .**

The *β*-*dual* and *γ*-*dual* of *h*(*gI*) are *σ*(*l*_*∞*_(*gI*)).

**Proof**

Let $\tilde{u}=({\tilde{u}}_{k})\in h(gI)$ and $\left({\tilde{v}}_{k})\in \sigma (l_{\infty }(gI)\right)$. Since $\left({\tilde{u}}_{k})\in h(gI\right)$, we have $\lim_{k\to \infty }d[{\tilde{u}}_{k},\tilde{0}]=0$. Therefore for given *ε* > 0, there exists *n*_0_ such that $d[{\tilde{u}}_{k},\tilde{0}]<\varepsilon$.

Since $\left({\tilde{v}}_{k})\in \sigma (l_{\infty }(gI)\right)$, $\sup_{k}d[{\left(B\tilde{v}\right)}_{k},\tilde{0}]<\infty$. Thus $d[{\tilde{v}}_{k},\tilde{0}]<\infty$ for all *k* and *n*.

Hence there exists an *M* > 0 such that $d[{\tilde{v}}_{k},\tilde{0}]<M$ for all *k* and *n*.

Now,

(29) $$\begin{array}{c} d\left[{\tilde{u}}_{k}{\tilde{v}}_{k},\tilde{0}\right]<d\left[{\tilde{u}}_{k},\tilde{0}\right]d\left[{\tilde{v}}_{k},\tilde{0}\right]<\varepsilon M. \end{array}$$

$\sum_{k=1}^{\infty }{\tilde{u}}_{k}{\tilde{v}}_{k}$ converges uniformly by Weierstrass *M*-test.

Thus

(30) $$\begin{array}{c} \sigma \left(l_{\infty }\left(gI\right)\right)\subset {\left[h\left(gI\right)\right]}^{\beta }. \end{array}$$

Conversely, suppose $({\tilde{v}}_{k})\in [h(gI){]}^{\beta }$. Then the series $\sum_{k=1}^{\infty }{\tilde{u}}_{k}{\tilde{v}}_{k}$ converges for all $\left({\tilde{u}}_{k})\in h(gI\right)$. This also holds for the sequence of modals $\left({\tilde{u}}_{k}\right)$ defined by $\left({\tilde{u}}_{k})=([-1,1]\right)$ for all *k* ∈ *N*.

$\sum_{k=1}^{\infty }{\tilde{u}}_{k}{\tilde{v}}_{k}=\sum_{k=1}^{\infty }[-1,1][{\underline{v}}_{k},{\overset{-}{v}}_{k}]=\sum_{k=1}^{\infty }\max\{\left|{\underline{v}}_{k}\right|,\left|{\overset{-}{v}}_{k}\right|]$ converges uniformly. Thus $\sup_{k}d[{\left(B\tilde{v}\right)}_{k},\tilde{0}]<\infty$.

Hence

(31) $$\begin{array}{c} \left({\tilde{v}}_{k}\right)\in \sigma \left(l_{\infty }\left(gI\right)\right),\;\;{\left[h\left(gI\right)\right]}^{\beta }\subset \sigma \left(l_{\infty }\left(gI\right)\right). \end{array}$$

From (30) and (31), [*h*(*gI*)]^*β*^ = *σ*(*l*_*∞*_(*gI*)). This completes the proof.

**Theorem 16 .**

Consider

(i) *h*(*gI*) ⊂ *l*_1_(*gI*)∩∫*c*_0_(*gI*).

(ii) *h*(*gI*) = *l*_1_(*gI*)∩∫*bv*(*gI*) = *l*_1_(*gI*)∩∫*bv*_0_(*gI*).

**Proof**

(i) Let $\tilde{u}=({\tilde{u}}_{k})\in h(gI)$. Then $\sum_{k=1}^{\infty }d((T\tilde{u}{)}_{k},\tilde{0})<\infty$ and $\lim_{k\to \infty }d[{\tilde{u}}_{k},\tilde{0}]=0$.

Consider $\sum_{k=1}^{\infty }d({\tilde{u}}_{k},\tilde{0})\leq \sum_{k=1}^{\infty }d((T\tilde{u}{)}_{k},\tilde{0})<\infty$. Therefore, $\tilde{u}=({\tilde{u}}_{k})\in l_{1}(gI)$.

And also since $\lim_{k\to \infty }d[k{\tilde{u}}_{k},\tilde{0}]=\lim_{k\to \infty }kd[{\tilde{u}}_{k},\tilde{0}]=0$, $\tilde{u}=({\tilde{u}}_{k})\in \int c_{0}(gI)$.

Therefore, $\tilde{u}=({\tilde{u}}_{k})\in l_{1}(gI)\cap \int c_{0}(gI)$. Hence *h*(*gI*) ⊂ *l*_1_(*gI*)∩∫*c*_0_(*gI*).

(ii) For *k* = 1,2,…,

(32) ku~k−u~k+1=u~k+1+ku~k−ku~k+1−u~k+1=u~k+1+ku~k−k+1u~k+1.

$\tilde{u}=({\tilde{u}}_{k})\in h(gI)$ implies

(33) ∞>∑k=1∞dTu~k,0~=∑k=1∞dku~k−u~k+1,0~≥∑k=1∞dku~k−k+1u~k+1,0~   −∑k=1∞du~k+1,0~.

The last series is convergent since *h*(*gI*) ⊂ *l*_1_(*gI*). Hence also $\sum_{k=1}^{\infty }d[k{\tilde{u}}_{k}-(k+1){\tilde{u}}_{k+1},\tilde{0}]<\infty$ and therefore *h*(*gI*) ⊂ ∫*bv*(*gI*).

Hence

(34) $$\begin{array}{c} h\left(gI\right)\subset l_{1}\left(gI\right)\cap \int bv\left(gI\right). \end{array}$$

Conversely, (32) implies for $({\tilde{u}}_{k})\in l_{1}(gI)\cap \int bv(gI)$

(35) ∑k=1∞dTu~k,0~=∑k=1∞dku~k−u~k+1,0~≤∑k=1∞dku~k−(k+1)u~k+1,0~ +∑k=1∞du~k+1,0~<∞,lim⁡k→∞du~k,0~=0.

Thus $\left({\tilde{u}}_{k}\right)\in h\left(gI\right)$.

Therefore,

(36) $$\begin{array}{c} l_{1}\left(gI\right)\cap \int bv\left(gI\right)\subset h\left(gI\right). \end{array}$$

Hence from (34) and (36), we have shown that *h*(*gI*) = *l*_1_(*gI*)∩∫*bv*(*gI*).

Similarly, we can prove other equalities.
